# Dose–Response Association between Physical Activity and Health-Related Quality of Life in General Population: A Population-Based Pooled Study

**DOI:** 10.3390/healthcare10081460

**Published:** 2022-08-03

**Authors:** Hosam Alzahrani

**Affiliations:** Department of Physical Therapy, College of Applied Medical Sciences, Taif University, Taif 21944, Saudi Arabia; halzahrani@tu.edu.sa

**Keywords:** health, HRQoL, physical activity, exercise, dose–response

## Abstract

Objective: The objective of this study was to examine the dose–response association between moderate-to-vigorous intensity physical activity (MVPA) and health-related quality of life (HRQoL) within the context of a large representative national survey from Wales, in the UK. Methods: Data for adults aged 16 years and older, living in Wales, UK, and participating in the Welsh Health Survey (WHS; 2011–2015) were employed. HRQoL was assessed using the 36-item short form (SF-36). Participants were categorized into six groups based on weekly minutes (min/week) of MVPA variable: none (no MVPA); very low (>0 to <90); low (≥90 to <150); sufficient (150 to <300); high (≥300 to <390); and very high (≥390). The association between MVPA and HRQoL was explored using multiple linear regression and generalized linear models. Results: Of the 74,578 adults in the survey cohort, 67,770 adults were included in the analyses. The results showed consistent direct curvilinear associations between MVPA and HRQoL for all SF-36 domains (*p* < 0.001), in both the minimally and maximally adjusted models; with the highest scores observed for those meeting the recommended physical activity guidelines (sufficient, high, and very high). The scores of the overall HRQoL and SF-36 domains demonstrated a consistent positive gradient from the very low level to the sufficient level, suggesting a dose–response relationship. Conclusions: The results showed a direct curvilinear association between MVPA and the overall HRQoL and SF-36 domains, with better scores observed for those meeting the recommended guidelines.

## 1. Introduction

Various adverse health consequences are associated with being physically inactive, including an increased risk of several illnesses, such as cardiovascular disease, respiratory disease, cancer, depression, and diabetes [1,2]. Recent estimates have indicated that the annual number of deaths due to health problems associated with physical inactivity is approximately 3.2 million, making it the fourth most important leading risk factor for mortality worldwide [3]. Engaging in physical activities not only prevents or delays the development of chronic diseases but also improves health-related quality of life (HRQoL) [4].

HRQoL is a useful indicator of health status, as it encompasses multiple domains related to health, including physical and psychosocial well-being [5,6]. HRQoL is an important multi-dimensional concept in health research and can assist in making clinical decisions for preventing and treating illnesses. Furthermore, HRQoL is associated with hospitalization and mortality in the general population [7,8,9].

A better understanding of factors or healthy lifestyles, such as engaging in physical activity that can affect HRQoL, might offer policymakers a basis to justify promoting physical activity in the general population. Although previous studies have demonstrated positive associations between physical activity and HRQoL, evidence is still limited in the general population, as these studies have focused on specific interventions or specific populations (e.g., elderly people and those with specific diseases) [10,11]. People with chronic diseases and those who are older have a lower HRQoL due to poor physical health and lack of balance and strength [12]; therefore, the results reported on the associations between physical activity and HRQoL in these populations might not be generalizable to adults and disease-free populations. Previous studies have focused on the association between physical activity and the prevalence of morbidity and mortality [13,14]. Therefore, these points might explain the limited evidence regarding the change in HRQoL attributable to engaging in physical activity [13,14].

The World Health Organization recommends that people should engage in at least 150 min of moderate-intensity physical activity per week, or at least 75 min of vigorous-intensity physical activity per week, or a combination of both moderate- and vigorous-intensity activity (MVPA) equivalent to 150 min per week for overall health [4,15,16]. Engaging in at least 300 min per week of moderate-intensity physical activity, or at least 150 min of vigorous-intensity physical activity is further recommended for additional health benefits [4,15,16]. Although these guidelines are widely recommended for improving general health, it is unclear whether they are applicable for improving HRQoL. Little is known about the optimal dose needed or the various amounts of physical activity associated with a better HRQoL in the general population [10]. Additionally, there remains limited evidence regarding whether these associations between physical activity and HRQoL would differ across sexes and different age groups. A previous review investigated the relationship between physical activity and HRQoL in the general population, and despite concluding a positive relationship, the authors stressed the importance of conducting further studies to understand the dose–response relationship and whether different modes of physical activity have a stronger positive association with HRQoL [10].

The present study aimed to explore the dose–response association between physical activity and HRQoL within the context of a large representative national survey in Wales, UK.

## 2. Methods

### 2.1. Study Design

This cross-sectional study used data from the Welsh Health Survey (WHS) (2011, 2012, 2013, 2014 and 2015), a large population-based survey [17]. These surveys involved 74,578 adults aged ≥16 years. The current study has been reported according to the Strengthening the Reporting of Observational Studies in Epidemiology (STROBE) guidelines [18].

The WHS is an annual health surveillance and national representative that measures the health status of the population living in Wales, UK. The WHS is a household-based survey that randomly recruited participants in multiple stages with probable stratified sampling at two stages: the first stage included selecting postcode sectors, and the second stage included selecting household addresses. The methods of data collection and sampling used in the surveys are described in detail elsewhere [19]. Ethics approval for the WHS was granted by the Ethics Committee of the National Center for Social Research, London, UK. The data were anonymized and made available to bona fide researchers via the UK Data Archive (Available online: http://data-archive.ac.uk/ (accessed on 1 April 2021)).

### 2.2. Physical Activity Variables

Participants reported the number of days in the previous week during which they engaged in at least 30 min of light-, moderate-, and vigorous-intensity activity/exercise (or segments of at least 10 min of activity on the same day that account for a total of 30 min) (Supplementary Materials Table S1). Light activity included walking at an average pace, golf, housework (e.g., hoovering or vacuuming, dusting), and light gardening (e.g., weeding). Moderate activity included fast walking, gentle swimming, dancing, heavy housework (e.g., walking with heavy shopping, spring cleaning), and heavy gardening (e.g., digging). Vigorous activity included aerobics, running, jogging, fast cycling, football, squash, and swimming lengths.

The MVPA variable was derived and calculated by multiplying the vigorous-intensity activity (min/week) by 2 and then adding this to the moderate-intensity activity (min/week). This calculation method for estimating the weekly minutes of MVPA was used in previous published population-based studies [11,20,21]. A six-level dose–response of weekly minutes of MVPA variable was then created: (1) none (not reporting any MVPA throughout the week), very low (>0 to <90), low (≥90 to <150), sufficient (150 to <300), high (≥300 to <390), and very high (≥390). These categories were created following the previous studies and to reflect the current physical activity guidelines [16,22]. Participants assigned to the sufficient or higher levels were considered to meet the current physical activity guidelines [16].

### 2.3. Health-Related Quality of Life

HRQoL was assessed using the 36-item short-form survey (SF-36). The SF-36 measures eight domains: physical functioning, role limitations due to physical health (role-physical), role limitations due to emotional problems (role-emotional), emotional well-being (mental health), energy/fatigue (vitality), bodily pain, social functioning, and general health perceptions. The total percentage was calculated for each domain (ranging from 0 [worst status] to 100 [best status]). The average of the eight domains was used to calculate the overall HRQoL (0–100), with a higher score indicating better health [23].

The SF-36 has been broadly validated for use in the general population as well as patient groups and can be easily administered by clinicians or patients at home [24,25,26,27,28,29]. More information on SF-36 can be found elsewhere [29,30].

### 2.4. Potential Confounders

The following possible confounding factors were identified from existing literature [31,32,33,34,35,36,37,38,39]. These factors were included in the analyses, and they were as follows: sex; age; body mass index (BMI) (underweight [<18.5 kg/m^2^], normal weight [18.5–24.9 kg/m^2^], overweight [25.0–29.9 kg/m^2^], and obesity condition [≥30.0 kg/m^2^]); education (tertiary degree or above, other qualifications, or no education qualification); employment status (currently employed or unemployed); smoking status (current smoker, never smoker, or previous smoker); mental illness (yes/no), musculoskeletal disorders (yes/no); and the presence of chronic illness (e.g., existing heart or respiratory condition, stroke, cancer, or diabetes). The data obtained on chronic illnesses in the WHS were collected by questioning the participants in regard to whether they had ever been or are currently being treated for any of the chronic illnesses.

### 2.5. Statistical Analyses

The main analysis in this study was performed to examine the relationship between MVPA and HRQoL. Subsequently, a secondary analysis was conducted to examine the relationship between MVPA and each domain of the SF-36. The analyses were performed using generalized linear models and multiple linear regression to detect linear trend *p*-values. Different models were adjusted for and included in the analyses: (1) sex and age; (2) and additionally for BMI, education level, employment, smoking status, mental illness, musculoskeletal disorder, chronic illnesses, and light-intensity physical activity. Generalized linear model coefficients indicate mean differences in HRQoL between the reference group (none) and each of the other MVPA groups.

In addition, the analyses were stratified by age (early adulthood: 20–39 years, middle adulthood: 40–59 years, late adulthood: ≥60 years of age) and sex (male and female) [40].

The association between physical activity and HRQoL could be clinically important. A minimal clinical important difference (MCID) was defined by Jaeschke, Singer [41] as “The smallest difference in score in the domain of interest which patients perceive as beneficial and which would mandate, in the absence of troublesome side effects and excessive cost, a change in the patient’s management”. The results from the generalised linear models analyses were compared with MCID for the change in each domain or the overall HRQoL scores from performing MVPA. An MCID has been founded for the SF-36 on the basis of statistical grounds (≥5 points) [42,43]. Therefore, differences in SF-36 scores of 5 points or more were reported as clinically meaningful.

The analyses were performed in March 2022 using IBM SPSS Statistics version 22. For all statistical tests, a *p*-value of <0.05 was considered statistically significant.

## 3. Results

### 3.1. Descriptive Results

The general characteristics of the participants are presented in Table 1. Of the 74,578 adults in the survey cohort, 67,770 adults were included in the analyses. Approximately 37.0% of the participants were aged ≥60 years, 53.6% were females, 35.4% had normal weight, 48.8% never smoked, 58.4% had an education qualification lower than a tertiary degree, and 48.7% were unemployed. Most participants had at least one chronic illness (51.9%), 84.6% had no mental illness, 63.2% had no musculoskeletal disorder, and 43.3% did not participate in any MVPA throughout the week. Missing data in the demographic variables were reported in the Supplementary Materials Table S2.

### 3.2. Associations between Physical Activity and HRQoL

**Figure 1:**
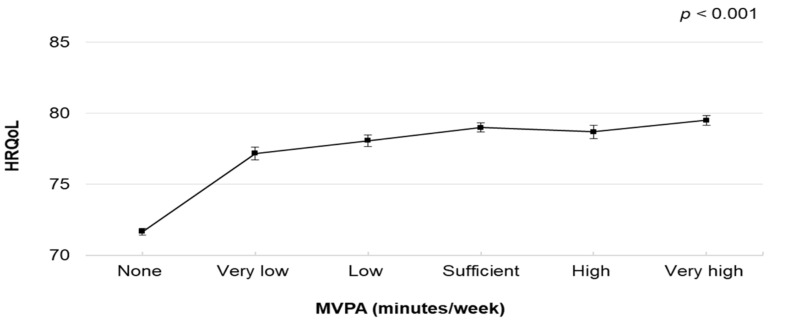
Multivariable-adjusted means and 95% Cis of HRQoL by MVPA volume (None: not reporting any MVPA; Very low: reporting >0–<90 min/week; Low: reporting ≥90–<150 min/week; Sufficient: reporting 150–<300 min/week; High: reporting ≥300–<390; Very high: reporting ≥390). The model was adjusted for age, sex, body mass index, education, employment, smoking status, mental illness, musculoskeletal disorder, chronic illness (including any heart and respiratory conditions, diabetes and cancer) and light-intensity physical activity. Abbreviations: Cis, confidence intervals; HRQoL, health-related quality of life; MVPA, moderate- to vigorous-intensity physical activity.

The associations between MVPA and HRQoL are presented in Table 2. The dose–response associations are illustrated in Figure 1. The multivariable-adjusted linear regression (model 2) showed a significant curvilinear association between weekly minutes of MVPA and overall HRQoL (Figure 1). The difference in the score of the overall HRQoL by the MVPA level was attenuated after adjustment for all confounding factors (model 2); however, the significant association persisted (Table 2). Compared with those who did not participate in any MVPA, those classified as “very high” had a higher HRQoL (coefficient = 7.84; 95% confidence interval [CI], 7.39–8.29), followed by “sufficient” (coefficient = 7.33; 95% CI, 6.91–7.75) and “high” (coefficient = 7.03; 95% CI, 6.48–7.58).

**Figure 2:**
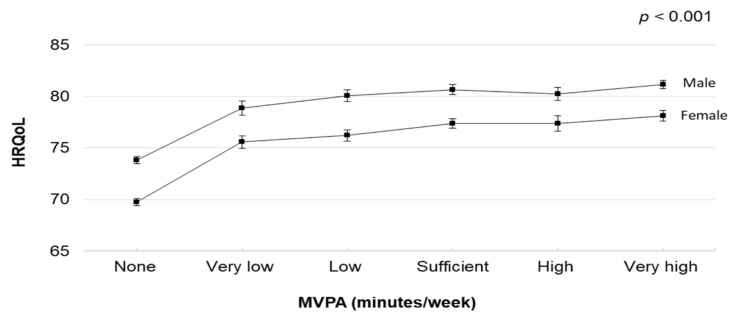
Multivariable-adjusted means and 95% Cis of HRQoL by MVPA volume (None: not reporting any MVPA; Very low: reporting >0–<90 min/week; Low: reporting ≥90–<150 min/week; Sufficient: reporting 150–<300 min/week; High: reporting ≥300–<390; Very high: reporting ≥390). The model was adjusted for age, body mass index, education, employment, smoking status, mental illness, musculoskeletal disorder, chronic illness (including any heart and respiratory conditions, diabetes, and cancer), and light-intensity physical activity. Abbreviations: Cis, confidence intervals; HRQoL, health-related quality of life; MVPA, moderate- to vigorous-intensity physical activity.

**Figure 3:**
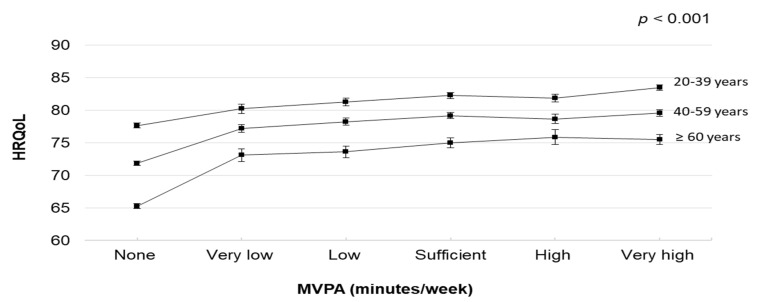
Multivariable-adjusted means and 95% Cis of HRQoL by MVPA volume (None: not reporting any MVPA; Very low: reporting >0–<90 min/week; Low: reporting ≥90–<150 min/week; Sufficient: reporting 150–<300 min/week; High: reporting ≥300–<390; Very high: reporting ≥390). The model was adjusted for sex, body mass index, education, employment, smoking status, mental illness, musculoskeletal disorder, chronic illness (including any heart and respiratory conditions, diabetes, and cancer), and light-intensity physical activity. Abbreviations: Cis, confidence intervals; HRQoL, health-related quality of life; MVPA, moderate- to vigorous-intensity physical activity.

The dose–response relationships between MVPA and HRQoL in the stratified analyses by sex and age are illustrated in Figure 2 and Figure 3 (and Supplementary Materials Table S3). In the stratified analyses by sex, MVPA was associated with HRQoL. Male and female participants who engaged in very high MVPA had higher HRQoL scores (predicted marginal means 81.16 [95% CI, 80.74–81.58] and 78.11 [95% CI, 77.58–78.63], respectively). In the stratified analyses by age, MVPA was associated with HRQoL. Participants in their early and middle adulthood who engaged in very high MVPA had higher HRQoL scores (predicted marginal means 83.52 [95% CI, 83.01–84.04] and 79.59 [95% CI, 79.07–80.12], respectively). However, participants in their late adulthood who engaged in high MVPA had higher HRQoL scores (predicted marginal mean 75.86 [95% CI, 74.72–77.01]).

**Figure 4:**
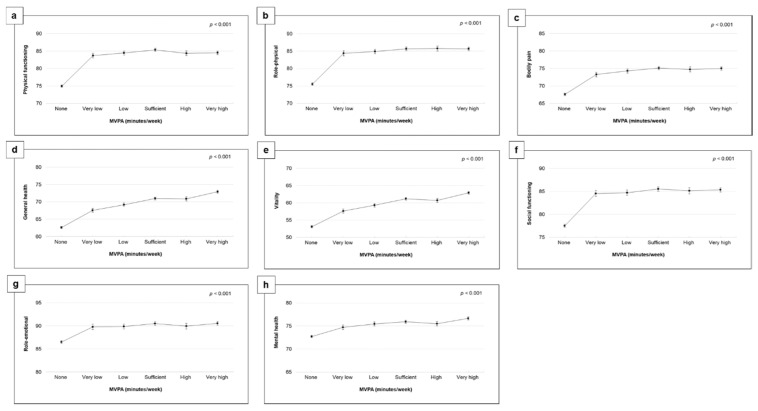
Multivariable-adjusted means and 95% Cis of HRQoL by MVPA volume (None: not reporting any MVPA; Very low: reporting >0–<90 min/week; Low: reporting ≥90–<150 min/week; Sufficient: reporting 150–<300 min/week; High: reporting ≥300–<390; Very high: reporting ≥390). The model was adjusted for age, sex, body mass index, education, employment, smoking status, mental illness, musculoskeletal disorder, chronic illness (including any heart and respiratory conditions, diabetes, and cancer), and light-intensity physical activity. (**a**) Physical functioning, (**b**) Role-physical, (**c**) Bodily pain, (**d**) General health, (**e**) Vitality, (**f**) Social functioning, (**g**) Role-emotional, (**h**) Mental health. Abbreviations: CIs, confidence intervals; HRQoL, health-related quality of life; MVPA, moderate- to vigorous-intensity physical activity.

Consistent direct associations were demonstrated between MVPA and SF-36 domains, although adjustments for all confounding factors attenuated these associations (Table 2 and Figure 4). The multivariable-adjusted linear regression (model 2) revealed curvilinear associations between MVPA and SF-36 domains (Figure 4). The strongest associations (a difference of up to 9–10 points across the MVPA levels) were observed for physical functioning, role-physical, vitality, and general health. Furthermore, engaging in any MVPA level was associated with better scores on all SF-36 domains compared with those who did not engage in any MVPA.

## 4. Discussion

The results of this study found a direct curvilinear association between weekly minutes of MVPA and the overall HRQoL; all participants who engaged in any level of MVPA had better overall HRQoL scores than those who did not engage in any MVPA. More specifically, participants who met the recommended physical activity guidelines had higher HRQoL scores. These associations extend across all age groups and sexes. To the best of our knowledge, this is the first population-based study to investigate the nature of the dose–response association between MVPA and HRQoL. The scores of the overall HRQoL and SF-36 domains demonstrated a consistent positive gradient from the very low level to the sufficient level, suggesting a dose–response relationship. This is a novel finding that merits attention. Future prospective studies are required to confirm these findings.

Regarding variations of minimal clinical importance, the analyses demonstrated that participating in any MVPA resulted in statistically significant and clinically important variations in overall HRQoL and SF-36 domains scores, apart from role-emotional and mental health. It may be that a large proportion of individuals are not capable of increasing their physical activity, but the independent nature of the effect gives a strong indication that clinically significant alterations can be achieved for those who can.

The findings of this study complement those of previous studies that demonstrated a positive association between physical activity and HRQoL [10]. In the current study, those who engaged in high levels of MVPA (sufficient, high, and very high levels) had the best scores on HRQoL and SF-36 domains. A similar association was observed by Brown, Balluz [44], who found that meeting the recommended physical activity guidelines was associated with better HRQoL. These results suggest that meeting the recommended physical activity guidelines might also be associated with HRQoL and not only with objective health outcomes. As it is highly beneficial to health to achieve physical activity recommendations, the results of this study are important in terms of encouraging physical activity for the general population. Many individuals fail to satisfy the physical activity recommendations [45]. However, our results showed that relatively low MVPA levels might also be beneficial for HRQoL. Therefore, future research should find effective ways to motivate people to engage in physical activity.

Important differences have been observed in the HRQoL scores between MVPA categories across age groups (2.57–5.98 points in early adults, 5.35–7.74 points in middle adults, and 7.85–10.23 points in late adults) and different sexes (5.03–7.35 points in men and 5.85–8.38 points in women). These results showed that the observed differences in HRQoL associated with MVPA differed according to sociodemographic variables. These findings are consistent with the results of previous studies that found important differences in the HRQoL scores between physical activity levels across different age and sex groups [44,46]. Brown, Balluz [44] reported that the strong association between physical activity and HRQoL was observed in adults aged ≥45 years, which may align with the results of the present study, which found that this association tended to be stronger in late adults (≥60 years). Furthermore, Tessier, Vuillemin [47] reported that the association tended to be stronger in women than in men, which is consistent with the findings of the current study. These observed differences in HRQoL are of importance to policy-makers and relevant to public health, as they may have a considerable impact at the societal level [48].

The results demonstrated consistent curvilinear associations between MVPA and all SF-36 domains. More specifically, sufficient and very high levels were associated with the highest scores for all SF-36 domains (except role-physical, wherein those classified as having sufficient and high levels achieved the highest scores). Furthermore, the strongest associations between MVPA and SF-36 domains (a difference of up to 8–10 points across the MVPA levels) were observed for physical functioning, role-physical, vitality, social functioning, and general health. This finding aligns with the results of previously published studies that also found that those meeting the recommended physical guidelines reported higher scores in the physical functioning, vitality, and general health domains [49,50,51]. These results indicate that there is almost no agreement among studies that the physical components of HRQoL are more closely associated with physical activity than the mental components. Further prospective studies are required to better understand the nature of this association.

The results of the present study might not be directly comparable with those of previous studies due to the differences in the assessment and categorization of physical activity and HRQoL, as well as the inclusion of different types of physical activities among studies. Furthermore, most previous studies did not adjust for several HRQoL determinants (e.g., mental illness, musculoskeletal disorders, and chronic illnesses), as conducted in the current study [10].

One of the strengths of the current study is that it used large and multiple pooled samples comprising a series of nationally representative samples drawn from the general population living in Wales. The larger the population samples, the more likely it is to have sufficient variability in the exposure and outcome, making it possible to identify relationships when they exist. Another strength is the availability of sufficient data on comorbidities (chronic diseases, musculoskeletal disorders, and mental illness), which enabled us to adjust for the presence of confounding factors that might confound the association between physical activity and HRQoL.

Limitations of the present study include the use of self-administered questionnaires for assessing physical activity and HRQoL. Using self-reported questionnaires may result in recall bias, which could cause an inaccurate estimation in either the direction or misclassification of the level of physical activity attained or the HRQoL score [52]. This inaccurate estimation, however, might be alleviated to some degree by the good reliability, discriminant validity, and convergent validity demonstrated by the SF-36 tool [24,25,26,27,28,29,53]. Furthermore, a previous study found that people classified as being physically active by objective measures had a similar mean HRQoL value (0.918) to those classified as physically active by self-reports (0.916) [54]. Another limitation is the inability to control for some factors including occupational physical activity, sedentary behavior, and sleep, as there is limited availability of data in the survey on these factors.

## 5. Conclusions

The results showed a direct curvilinear association between MVPA and overall HRQoL and SF-36 domains, with better scores observed for those meeting the recommended guidelines. Furthermore, the scores of the overall HRQoL and SF-36 domains demonstrated a consistent positive gradient from the very low level to the sufficient level, suggesting a dose–response relationship. Future prospective and interventional studies targeting the general population are required to confirm the results of the current study and to provide a better understanding of the cause–effect relationship between physical activity and HRQoL.

## Figures and Tables

**Table 1:** Baseline characteristics of participants recruited from the Welsh Health Survey (2011, 2012, 2013, 2014, 2015).

Characteristics	Total No. (%)
**Age (years)**	
16–39	22,037 (29.5)
40–59	24,911 (33.4)
≥60	27,630 (37.0)
**Gender**	
Male	34,587 (46.4)
Female	39,991 (53.6)
**BMI, kg/m^2^**	
Underweight (<18.5)	1367 (1.8)
Normal (18.5 to 24.9)	26,409 (35.4)
Overweight (25 to 30)	24,942 (33.4)
Obesity condition (≥30)	15,820 (21.2)
**Smoking status**	
Never smoker	36,390 (48.8)
Previous smoker	22,280 (29.9)
Current smoker	14,857 (19.9)
**Highest level of education**	
Degree or above	11,637 (15.6)
Other qualifications	43,533 (58.4)
No qualification	14,094 (18.9)
**Employment status**	
Currently employed	34,955 (46.9)
Not employed	36,347 (48.7)
**Prevalent chronic illness**	
No	34,547 (46.3)
Yes	38,671 (51.9)
**Prevalent mental illness**	
No	63,078 (84.6)
Yes	8454 (11.3)
**Prevalent musculoskeletal disorder**	
No	47,104 (63.2)
Yes	24,150 (32.4)
**MVPA (min/week)**	
None (0)	32,398 (43.3)
Very low (0–<90)	5437 (7.3)
Low (90–<150)	7250 (9.7)
Sufficient (150–<300)	11,208 (15.0)
High (300–<390)	5273 (7.1)
Very high (≥390)	13,012 (17.4)

Abbreviations: BMI, body mass index; MVPA, moderate- to vigorous-intensity physical activity.

**Table 2:** Multivariable-adjusted associations between MVPA, and HRQoL and SF-36 domains in general population.

	Model 1 ^a^	Model 2 ^a^
	Coefficient (95% CI)	Coefficient (95% CI)
**HRQoL ^b^**		
None	Referent	Referent
Very low	10.821 (10.255, 11.387)	5.52 (5.00, 6.04)
Low	12.816 (12.313, 13.319)	6.40 (5.93, 6.88)
Sufficient	14.264 (13.837, 14.692)	7.33 (6.91, 7.75)
High	14.514 (13.940, 15.088)	7.03 (6.48, 7.58)
Very high	15.523 (15.113, 15.932)	7.84 (7.39, 8.29)
Trend *p* value	<0.001	<0.001
R^2^	0.182	0.418
***SF-36 domains***		
**Physical functioning ^b^**		
None	Referent	Referent
Very low	16.50 (15.73, 17.26)	8.75 (8.01, 9.49)
Low	18.70 (18.02, 19.37)	9.52 (8.85, 10.20)
Sufficient	20.33 (19.75, 20.90)	10.44 (9.84, 11.04)
High	20.22 (19.44, 20.99)	9.43 (8.65, 10.22)
Very high	20.66 (20.11, 21.21)	9.57 (8.93, 10.21)
Trend *p* value	<0.001	<0.001
R^2^	0.269	0.393
**Role-physical ^b^**		
None	Referent	Referent
Very low	15.90 (15.09, 16.72)	8.81(8.01, 9.61)
Low	17.92 (17.20, 18.64)	9.34 (8.61, 10.07)
Sufficient	19.32 (18.71, 19.94)	10.11 (9.46, 10.76)
High	20.43 (19.60, 21.26)	10.21 (9.36, 11.06)
Very high	20.76 (20.18, 21.35)	10.08 (9.39, 10.77)
Trend *p* value	<0.001	<0.001
R^2^	0.226	0.361
**Role-emotional ^b^**		
None	Referent	Referent
Very low	9.58 (8.84, 10.31)	3.28 (2.60, 3.96)
Low	10.89 (10.24, 11.54)	3.38 (2.77, 4.00)
Sufficient	12.04 (11.48, 12.59)	4.00 (3.45, 4.54)
High	12.47 (11.72, 13.22)	3.44 (2.72, 4.15)
Very high	13.16 (12.63, 13.69)	4.03 (3.45, 4.62)
Trend *p* value	<0.001	<0.001
R^2^	0.068	0.297
**Vitality ^b^**		
None	Referent	Referent
Very low	8.37 (7.75, 8.99)	4.57 (3.92, 5.21)
Low	10.80 (10.25, 11.35)	6.24 (5.65, 6.83)
Sufficient	13.17 (12.70, 13.64)	8.09 (7.57, 8.61)
High	12.86 (12.23, 13.49)	7.64 (6.96, 8.33)
Very high	15.39 (14.95, 15.84)	9.86 (9.30, 10.42)
Trend *p* value	<0.001	<0.001
R^2^	0.113	0.265
**Mental health ^b^**		
None	Referent	Referent
Very low	6.03 (5.47, 6.59)	1.98 (1.43, 2.54)
Low	7.49 (6.99, 7.98)	2.71 (2.20, 3.22)
Sufficient	8.32 (7.90, 8.74)	3.21 (2.76, 3.66)
High	8.20 (7.63, 8.77)	2.77 (2.18, 3.36)
Very high	9.33 (8.93, 9.74)	3.92 (3.44, 4.40)
Trend *p* value	<0.001	<0.001
R^2^	0.049	0.261
**Social functioning ^b^**		
None	Referent	Referent
Very low	14.17 (13.40, 14.95)	7.05 (6.30, 7.81)
Low	15.87 (15.18, 16.56)	7.23 (6.54, 7.91)
Sufficient	17.48 (16.90, 18.07)	8.02 (7.41, 8.63)
High	18.08 (17.29, 18.86)	7.65 (6.85, 8.45)
Very high	18.45 (17.89, 19.01)	7.85 (7.20, 8.50)
Trend *p* value	<0.001	<0.001
R^2^	0.113	0.311
**Bodily pain ^b^**		
None	Referent	Referent
Very low	10.63 (9.87, 11.40)	5.69 (4.91, 6.46)
Low	12.41(11.73, 13.08)	6.72 (6.02, 7.43)
Sufficient	14.01(13.44, 14.59)	7.56 (6.94, 8.19)
High	13.88 (13.10, 14.65)	7.17 (6.35, 7.99)
Very high	14.50 (13.95, 15.05)	7.42 (6.75, 8.09)
Trend *p* value	<0.001	<0.001
R^2^	0.148	0.290
**General health ^b^**		
None	Referent	Referent
Very low	10.10 (9.45, 10.74)	4.96 (4.32, 5.60)
Low	12.57 (11.99, 13.14)	6.55 (5.96, 7.13)
Sufficient	14.98 (14.49, 15.46)	8.34 (7.83, 8.86)
High	15.09 (14.43, 15.75)	8.24 (7.56, 8.92)
Very high	17.22 (16.75, 17.68)	10.31 (9.76, 10.87)
Trend *p* value	<0.001	<0.001
R^2^	0.146	0.335

Abbreviations: CI, confidence interval; HRQoL, health-related quality of life; MVPA, moderate- to vigorous-intensity physical activity. Scale range for HRQoL and each SF-36 domain: 0–100, higher scores indicative of better status or health. None: not reporting any MVPA; Very low: reporting >0 –<90 min/week; Low: reporting ≥ 90–<150 min/week; Sufficient: reporting 150–<300 min/week; High: reporting ≥300–<390; Very high: reporting ≥390. ^a^ Model 1: adjusted for age and sex; Model 2: further adjustment for body mass index, education, employment, smoking status, mental illness, musculoskeletal disorder, chronic illness (including any heart and respiratory conditions, diabetes and cancer) and light-intensity physical activity. ^b^ Generalised linear model coefficients; coefficients indicate mean differences (in HRQoL and SF-36 domains) between the reference category (None) and each of the other MVPA groups, e.g., a value of 3 indicates that a specific category had a mean score that is 3 units higher than the referent group.

## Data Availability

The data were anonymized and made available to bona fide researchers via the UK Data Archive (Available online: http://data-archive.ac.uk/ (accessed on accessed on 1 April 2021)).

## Supplementary Materials

The following supporting information can be downloaded at: https://www.mdpi.com/article/10.3390/healthcare10081460/s1, Table S1: Physical activity questions used in the Welsh Health Survey; Table S2: Missing data in the demo-graphic variables; Table S3: Multivariable-adjusted means of HRQoL by MVPA volume, stratified by sex and age.
