# Supplementary material for: Dose–Response Association between Physical Activity and Health-Related Quality of Life in General Population: A Population-Based Pooled Study
Source: Healthcare (Basel). 2022 Aug 3;10(8):1460. doi: 10.3390/healthcare10081460 (PMC9408149; doi:10.3390/healthcare10081460)
Supplement: Supplementary file 1 [file healthcare-10-01460-s001.zip › healthcare-1801206-supplementary.pdf]

Supplementary Materials

Table S1 Physical activity questions used in the Welsh Health Survey.

EXERCISE

43a During the **7 days ending yesterday**, on which days did you do **LIGHT** exercise or physical activity **for at least 30 minutes**?
Blocks of activity lasting at least 10 minutes, which were done on the same day, can be counted towards the full 30 minutes.
Include physical activity which is part of your job

exltnum...exltmon, exlttue, exltwed, exltthu, exltfri, exltsat, exltsun

Tick all days that apply

	Mon	Tue	Wed	Thu	Fri	Sat	Sun	
**Light exercise / activity**<br>***For example*** Housework (eg hoovering, dusting), walking at an average pace, golf, light gardening (eg weeding)	☐ 01	☐ 02	☐ 03	☐ 04	☐ 05	☐ 06	☐ 07	2451-64
If no light exercise in the **last 7 days**, please tick	☐ 08 exltno							

43b During the **7 days ending yesterday**, on which days did you do **MODERATE** exercise or physical activity **for at least 30 minutes**?
Blocks of activity lasting at least 10 minutes, which were done on the same day, can be counted towards the full 30 minutes.
Include physical activity which is part of your job

exmodnum...exmodmon, exmodtue, exmodwed, exmodthu, exmodfri, exmodsat, exmodsun

Tick all days that apply

	Mon	Tue	Wed	Thu	Fri	Sat	Sun	
**Moderate exercise / activity**<br>***For example*** Heavy housework (eg spring cleaning, walking with heavy shopping), fast walking, dancing, gentle swimming, heavy gardening (eg digging)	☐ 01	☐ 02	☐ 03	☐ 04	☐ 05	☐ 06	☐ 07	2465-78
If no moderate exercise in the **last 7 days**, please tick	☐ 08 exmodno							

43c During the **7 days ending yesterday**, on which days did you do **VIGOROUS** exercise or physical activity **for at least 30 minutes**?
Blocks of activity lasting at least 10 minutes, which were done on the same day, can be counted towards the full 30 minutes.
Include physical activity which is part of your job

exvignum ...exvigmon, exvigtue, exvigwed, exvigthu, exvigfri, exvigsat, exvigsun

Tick all days that apply

	Mon	Tue	Wed	Thu	Fri	Sat	Sun	
**Vigorous exercise / activity**<br>***For example*** Running, jogging, squash, swimming lengths, aerobics, fast cycling, football	☐ 01	☐ 02	☐ 03	☐ 04	☐ 05	☐ 06	☐ 07	2479-92
If no vigorous exercise in the **last 7 days**, please tick	☐ 08 exvigno							

Table S2. Missing data in the demographic variables.

Characteristics	Total No. (%)	Missing data No. (%)
**Age (years)**	74,578 (100)	0 (0)
**Gender**	74,578 (100)	0 (0)
**BMI, kg/m^2**	68,538 (91.9)	6040 (8.1)
**Smoking status**	73,527 (98.6)	1051 (1.4)
**Highest level of education**	69,264 (92.9)	5314 (7.1)
**Employment status**	71,303 (95.6)	3275 (4.4)
**Prevalent chronic illness**	73,218 (98.2)	1360 (1.8)
**Prevalent mental illness**	71,532 (95.9)	3046 (4.1)
**Prevalent musculoskeletal disorder**	71,254 (95.5)	3324 (4.5)

Abbreviations: BMI, body mass index.

Table S3. Multivariable-adjusted means of HRQoL by MVPA volume, stratified by sex and age.

	Predicted marginal mean of HRQoL (95% CI) [a]	Coefficient (95% CI) [a]
**Sex**		
**Male**		
None	73.81 (73.47-74.16)	Referent
Very low	78.85 (78.16-79.54)	5.04 (4.27-5.80)
Low	80.08 (79.49-80.67)	6.27 (5.58-6.95)
Sufficient	80.67 (80.20-81.13)	6.85 (6.25-7.45)
High	80.23 (79.62-80.84)	6.42 (5.68-7.15)
Very high	81.16 (80.74-81.58)	7.35 (6.74-7.95)
Trend p value		<0.001
**Female**		
None	69.73 (69.40-70.06)	Referent
Very low	75.58 (74.96-76.19)	5.85 (5.14-6.55)
Low	76.21 (75.66-76.75)	6.48 (5.82-7.13)
Sufficient	77.36 (76.91-77.81)	7.63 (7.04-8.22)
High	77.36 (76.64-78.09)	7.63 (6.81-8.46)
Very high	78.11 (77.58-78.63)	8.38 (7.70-9.05)
Trend p value		<0.001
**Age (years)**		
**20-39**		
None	77.68 (77.22-78.13)	Referent
Very low	80.24(79.53-80.95)	2.56 (1.74-3.39)
Low	81.31 (80.70-81.93)	3.63 (2.87-4.40)
Sufficient	82.29 (81.82-82.77)	4.62 (3.94-5.30)
High	81.89 (81.19-82.59)	4.21 (3.34-5.08)
Very high	83.52 (83.01-84.04)	5.84 (5.08-6.60)
Trend p value		<0.001
**40-59**		
None	71.86 (71.45-72.26)	Referent
Very low	77.20 (76.47-77.94)	5.35 (4.51-6.18)
Low	78.25 (77.62-78.88)	6.39 (5.63-7.15)

Sufficient	79.20 (78.68-79.72)	7.35 (6.66-8.03)
High	78.69 (77.96-79.42)	6.84 (5.97-7.71)
Very high	79.60 (79.08-80.12)	7.74 (7.02-8.47)
Trend p value		<0.001
**≥ 60**		
None	65.27 (64.87-65.68)	Referent
Very low	73.12 (72.14-74.11)	7.85 (6.78-8.93)
Low	73.61 (72.75-74.48)	8.34 (7.36-9.32)
Sufficient	75.01 (74.28-75.74)	9.74 (8.87-10.60)
High	75.86 (74.72-77.01)	10.59 (9.35-11.84)
Very high	75.50 (74.75-76.25)	10.23 (9.32-11.14)
Trend p value		<0.001

Abbreviations: CIs, confidence intervals; HRQoL, health-related quality of life; MVPA, moderate- to vigorous-intensity physical activity.

Scale range for HRQoL and each SF-36 domain: 0-100, higher scores indicative of better status or health.

None: not reporting any MVPA; Very low: reporting > 0 – <90 min/week; Low: reporting ≥ 90 – <150 min/week; Sufficient: reporting 150 - <300 min/week; High: reporting ≥300 - <390; Very high: reporting ≥390).

[a] The model was adjusted for age, sex, body mass index, education, employment, smoking status, mental illness, musculoskeletal disorder, chronic illness (including any heart and respiratory conditions, diabetes and cancer) and light-intensity physical activity.
